# Concurrent acute myocardial infarction and acute ischemic stroke: Case reports and literature review

**DOI:** 10.3389/fcvm.2022.1012345

**Published:** 2022-11-01

**Authors:** Cheng-hong Bao, Chen Zhang, Xiao-min Wang, Yi-bin Pan

**Affiliations:** Department of Cardiology, Affiliated Jinhua Hospital, Zhejiang University School of Medicine, Jinhua, China

**Keywords:** myocardial infarction, acute ischemic stroke, intravenous thrombolysis, percutaneous coronary intervention, electrocardiogram (ECG)

## Abstract

Acute myocardial infarction (AMI) and acute ischemic stroke (AIS) are the main causes of disability and mortality worldwide. Although reperfusion therapy is the most effective treatment for the two diseases, it is still a great challenge for treating the two diseases at the same time. Here we share 2 cases: one patient was hospitalized for AMI, developed AIS after receiving percutaneous coronary intervention (PCI), and suffered from cardiac rupture after alteplase thrombolytic therapy. The other patient was admitted for AIS, who had sudden chest pain during the thrombolytic process of alteplase. Considering AMI, emergency PCI was performed, and he was finally discharged.

## Introduction

Cardiovascular and cerebrovascular diseases remain the leading cause of morbidity and mortality worldwide (1). Acute ischemic stroke (AIS) and acute myocardial infarction (AMI) are mutual risk factors, and both diseases should be timely diagnosed and treated. Sometimes we encounter patients with simultaneous AMI and AIS. As early as 2010, Omar et al. proposed the concept of cardiocerebral infarction (2). The incidence of acute cardiocerebral infarction is as low as 0.009% (3). AMI and AIS can occur at the same time or not (one disease precedes the other) (4). When the two diseases coexist, the condition worsens rapidly. Due to the different order of onset of the two diseases (simultaneous cardiocerebral infarction, AMI after AIS, AIS after AMI), different types of AMI and the length of onset time (within or beyond reperfusion time window), the optimal treatment plan and reperfusion method are still uncertain. Therefore, it brings great challenge and pressure to clinicians. We shared two cases to provide relevant evidence for clinical treatment decision-making.

## Case 1

**FIGURE 1:**
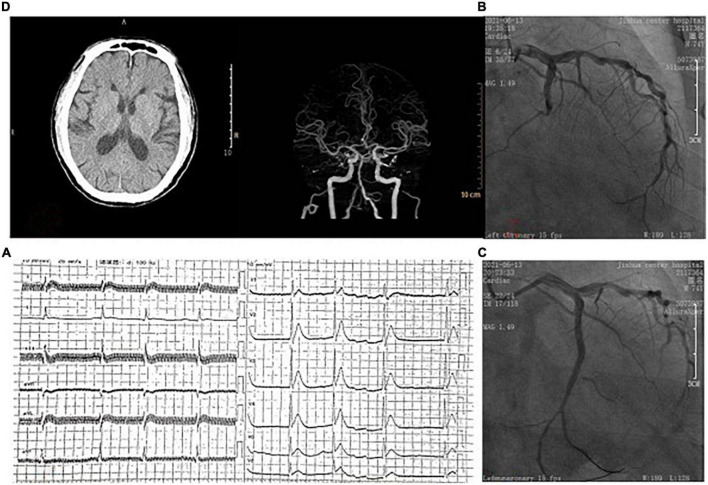
**(A)** On admission, ECG showed atrial fibrillation and ST-segment elevation in the anterior wall leads. **(B)** Coronary angiography showed occlusion of the left circumflex artery. **(C)** Blood flow was restored after coronary stent implantation. **(D)** Brain computed tomography showed no hemorrhage before intravenous thrombolytic therapy.

A 75-year-old male patient had a history of hypertension, diabetes, stroke and atrial fibrillation (AF), without oral anticoagulants. The patient was admitted to our emergency department due to chest pain for 12 h with heart rate of 90 beats/min and blood pressure (BP) of 120/70 mmHg. Emergency ECG showed AF, significant ST-T abnormality (Figure 1A). Transthoracic echocardiography showed left atrial enlargement, LVEF 48%, and minimal mitral regurgitation. AMI was diagnosed, and emergency coronary angiography (CAG) was performed to show diffuse plaque formation of right coronary artery (RCA), with 70% proximal stenosis, 90% middle stenosis, normal left main artery, 80% stenosis in the middle of left anterior descending artery (LAD), and 100% occlusion in the middle of left circumflex artery (LCX) (Figure 1B). LCX was considered as the culprit vessel, and finally a drug-eluting stent was implanted for LCX with Thrombolysis in myocardial infarction (TIMI) grade flow of 3 (Figure 1C). After percutaneous coronary intervention (PCI), the medicines of aspirin, clopidogrel, rivaroxaban, atorvastatin, metoprolol and irbesartan were used. Re-examination of the echocardiography showed reduced segmental movement of the left ventricular wall, LVEF 55%, no pericardial effusion, and no ventricular thrombosis. 10 days later, the patient had a sudden weakness of right limb and slurred speech. On physical examination, the right nasolabial fold became shallow, and the muscle strength of the right limb was grade 3. Emergency computed tomography angiography (CTA) of cerebral artery showed no obvious large vessel stenosis, no intracerebral hemorrhage (Figure 1D). AIS was considered after consultation by a neurologist. As the onset time was less than 4.5 h, intravenous recombinant tissue plasminogen activator (rt-PA) (0.9 mg/kg, total dose 70 mg, time 60 min) was performed. 40 min later, the patient suffered from a sudden drop in BP, unconsciousness, cardiac and respiratory arrest. Cardiopulmonary resuscitation was carried out immediately. Cardiac ultrasound showed a large amount of pericardial effusion. We did a pericardiocentesis and extracted 100 mL of blood fluid. But there were still no vital signs after active rescue. At last, the cause of death was considered to be cardiac rupture.

## Case 2

**FIGURE 2:**
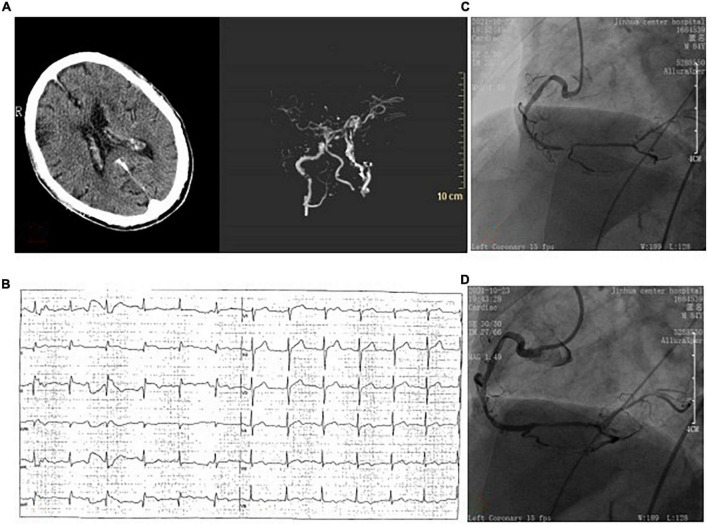
**(A)** Brain computed tomography angiography showed no hemorrhage before intravenous thrombolytic therapy, and showed weak imaging of bilateral anterior cerebral arteries. **(B)** ECG showed ST-segment elevation in the lower wall lead. **(C)** Coronary angiography showed 95% stenosis of the distal right coronary with thrombosis. **(D)** One drug-eluting stent was implanted in the right coronary artery.

An 84-year-old male patient with a history of cerebral infarction and hypertension was admitted to our emergency department on October 23, 2021. Before admission, the patient took aspirin, atorvastatin calcium tablets, and nifedipine controlled-release tablets orally. She suffered a slurred speech accompanied by right limb weakness for 1 h. Neurological examination indicated that the strength of right muscle was grade 0. And the National Institutes of Health Stroke Scale (NIHSS) score was 14. Cranial vascular CTA showed weak imaging of bilateral anterior cerebral arteries (Figure 2A). Diagnosis from the neurologist of AIS was made. Since CTA did not show arterial thrombosis, thrombectomy was temporarily not feasible, and intravenous thrombolysis was selected (rt-PA, 0.9 mg/kg). Forty five minutes later, electrocardiograph monitor showed heart rate of 40 beats/min, BP 70/40 mmHg and ECG showed ST segment elevation of inferior wall leads (Figure 2B). Meanwhile the patient felt obvious chest pain. Finally, cardiologists considered acute inferior wall myocardial infarction, and emergency PCI was performed. CAG showed 70% stenosis in the proximal segment of LAD, 80% stenosis in the proximal segment of LCX, 40% stenosis in the proximal segment of RCA, and 95% stenosis in the distal segment of RCA showing thrombus shadow (Figure 2C). Considering the culprit vessel was the RCA, thrombus aspiration was performed, and a drug-eluting stent was implanted, with TIMI grade flow of 3 (Figure 2D). Oral aspirin, clopidogrel and rosuvastatin were used. 48 h later, head CT showed no bleeding, but with left frontotemporal parietal lobe and left paraventricular infarction. Echocardiography showed minimal tricuspid regurgitation but no thrombus. The patient was actually monitored for AF but this was not detected. The maximum values of cTnI and NT-proBNP was 75 ng/mL and 3,520 pg/mL, respectively. After 2 weeks of treatment, the patient was discharged without chest pain or arrhythmia, but with mixed aphasia and right limbs muscle strength grade 2 (NIHSS 8).

## Discussion

Studies have shown that AIS increases the risk of AMI and vice versa. In a retrospective study from 2000 to 2017, among 11,622,528 patients admitted to hospital for AMI, 1.6% (183,896) developed AIS within 24 h. Compared with non-AIS patients, patients with AMI-AIS underwent CAG less frequently (46.9 vs. 63.8%) and PCI was similar (22.7 vs. 41.8%) (5). In another retrospective study from 2003 to 2014, among 864,043 patients admitted to hospital with AIS, 1.6% (66,977) had AMI (79.5% NSTEMI and 20.5% STEMI) within 24 h. The in-hospital mortality of stroke patients with AMI was higher (21.4 vs. 7.1%), and the length of hospital stay and treatment cost increased. Although CAG and PCI can reduce the mortality of patients with AMI-AIS, only 7.5 and 2% of patients received the above treatment, respectively (6). The main reason is that clinicians are concerned about related bleeding complications after interventional therapy, especially for patients with AIS after thrombolytic therapy. Meanwhile, clinical data and relevant guidelines are lacking. Ischemic stroke is one of the serious complications after AMI. The first month after AMI is considered to be the high-risk period of ischemic stroke (7). The 1-year mortality of AMI patients with stroke (51.5%) was 15% higher than that of AMI patients without stroke (37.1%) (8). Independent risk factors for AIS in AMI patients include age, female, history of stroke, diabetes, AF, heart failure, STEMI, CABG, etc. (9). At present, ventricular thrombosis is considered to be the main cause of stroke after AMI, and the local movement disorder of ventricular wall after AMI, blood stasis, inflammation and hypercoagulability are the main cause of ventricular thrombosis (10). However, it is unclear that how to prevent ventricular thrombosis in AMI patients without AF. According to U.S. guidelines, anticoagulation therapy for 3 months may be considered in STMI patients with reduced anterior wall movement or dyskinesia (11). In a Meta-analysis, the addition of oral anticoagulants to antiplatelet agents reduced the risk of cardiovascular death, myocardial reinfarction, and stroke in STEMI patients. But the benefit was not significant in NSTEMI patients (12). As is known, AF is an independent risk factor for stroke. For patients with AF complicated with AMI, triple antithrombotic therapy is currently recommended to reduce the incidence of stroke, and the specific duration of antithrombotic therapy is determined by ischemia-hemorrhage score (13). Interestingly, although PCI can reduce the incidence of AIS in patients with AMI (9), another rare cause of AIS in patients with AMI is PCI or CABG, with an incidence of 0.38%, which generally occurs in the perioperative period and is most common within 24 h after surgery. It is related to the plaque rupture of subclavian artery, aortic arch, carotid artery and other main arteries, and the thrombosis of guide wire and catheter tip (5, 14). The occurrence time of AIS in Case 1 was 10 days after PCI, but it would be difficult to say that the AIS in Case 1 was not related to PCI after 10 days, because previous studies have shown that there is a heightened risk of stroke even up to several months after PCI (15). Since the patient had AF, it is also possible that the stroke may be caused by shedding of thrombosis in the heart. The pathophysiological mechanism of AMI in AIS patients has been found to be higher prevalence of coronary heart disease and more cardiovascular risk factors. After stroke, autonomic nerve dysfunction and increased catecholamine hormone secretion lead to potential aggravation of coronary artery disease or stress myocardial injury (16). It was previously reported that a 41-year-old female patient was hospitalized for AIS with chest pain before thrombolytic therapy, which was considered to be complicated with acute anterior STMI. Thrombolytic therapy with alteplase was applied, but the patient suffered repeated chest pain, and emergency PCI was performed before she was eventually discharged (17).

The simultaneous occurrence of AMI and stroke is uncommon. Clinically, there are generally three types: the first is when AIS and AMI occur at the same time, the second is when AIS occurs after subacute myocardial infarction, and the third is that AMI occurs at the early stage of systemic thrombolysis in AIS (18). For heterochrony AMI and AIS, there is no doubt that dealing with the disease happened first, but at the same time, it will also bring some difficulty and relative contraindications to the treatment of subsequent diseases, such as ischemic stroke in the nearly past 3 months (excluding stroke within 4.5 h) is regarded as thrombolysis contraindications for AMI patients (19). AMI in the last 3 months is considered as a contraindication for thrombolytic therapy in AIS (20). Cardiac rupture and cardiac tamponade are the most serious complications of thrombolysis in AMI patients, with an incidence of 1–8% (21). Mannino et al. reported a case of acute anterior wall myocardial infarction after thrombolytic therapy in AIS patient, and finally suffered cardiac rupture and death. Meanwhile, the paper summarized 11 previously published cases of AMI after thrombolytic therapy in AIS patients, with a mortality rate as high as 64% (22). In this paper, stroke occurred after AMI in Case 1, and thrombolytic therapy was applied, resulting in complications of cardiac rupture and pericardial tamponade. The main cause of cardiac rupture is the dissolution of fibrin clots in the necrotic myocardial wall (18). There may have deficiencies in the management of Case 1. For patients with AIS of < 4.5 h duration, who used a NOAC during the last 48 h before stroke onset, intravenous thrombolysis is not suggested (23). We were unable to test for anti-Xa activity, and Andexanet alfa was not easily got. CTA showed no large vessel occlusion, so mechanical thrombectomy was not appropriate. Given the patient’s low dose of rivaroxaban, 2.5 mg twice a day, we speculated that the NOAC drug levels were low. The patient’s ischemic symptoms worsened. Finally, intravenous thrombolysis was performed. In Case 2, AMI occurred during thrombolytic period of stroke, and emergency PCI was executed due to hemodynamic instability and arrhythmia. Therefore, for patients with AMI during thrombolytic period of stroke, emergency PCI may bring benefits under the condition of hemodynamic instability.

For patients with concurrent or near concurrent AMI and stroke, there is still a lack of consensus on corresponding guidelines and treatment is very difficult. The mechanisms of simultaneous occurrence of cardiocerebral infarction are as follows (3, 24, 25). (1) Simultaneous thrombosis of coronary and cerebral arteries, such as AF, type I aortic dissection involving coronary artery and common carotid artery, electrical injury resulting in coronary and cerebral artery spasm, etc. (2) Stroke caused by heart disease, such as intraventricular thrombosis, patent foramina ovale (complicated with right heart infarction), and cardiac shock after AMI. (3) Cerebral-cardiac axis disorder or cerebral infarction lead to myocardial injury. The insular cortex plays an important role in the regulation of central autonomic nervous system. Pathological changes of insular cortex are related to AF, activation of cardiac sympathetic nerve, myocardial injury and interruption of the circadian rhythms of BP.

Previous published articles have shown that ST segment elevation of inferior wall lead is the most common ECG in patients with AMI-AIS, and the treatment and clinical prognosis vary from case to case. The case 2 also had inferior wall AMI, but due to the lack of large sample data, the correlation between inferior wall AMI and stroke cannot be explained at present (24). In the acute stage of stroke, troponin in some patients increases and ST-T changes occur in ECG (26), which brings difficulties to the diagnosis of AMI-AIS. Therefore, some scholars suggested that combined intravascular therapy could improve the diagnosis and success rate of AMI-AIS (3). Intravenous rt-PA thrombolytic therapy is a first-line therapy for stroke patients with onset less than 4.5 h (19). PCI is the first-line treatment for AMI, but for hospitals without PCI capability and STEMI within 12 h of onset, thrombolytic therapy is an alternative, while NSTEMI is not suitable for thrombolytic therapy (20). Kijpaisalratana et al. proposed a management method based on hemodynamic state, and named “hyperacaute simultaneous cardiocerebral infarction” for the patients with onset less than 4.5 h. For patients with cardiocerebral infarction with hemodynamic instability, emergency PCI was performed first, followed by intravascular treatment for AIS with large vascular occlusion. For patients with stable hemodynamics, rt-PA thrombolytic therapy was selected according to the standard dose of stroke, followed by vascular therapy for AIS and PCI for AMI according to the situation (24). The 2013 AHA/ASA guidelines recommended that thrombolytic therapy may be beneficial for patients with AMI-AIS within 3–4.5 h, and rt-PA is the only one recommended for stroke (27). However, the dose and duration of rt-PA are still controversial. Some scholars suggested that for patients with AMI-AIS within 4.5 h of onset, especially for patients of anterior wall AMI with reduced LVEF, rt-PA can be given according to the doses of STEMI thrombolysis, followed by PCI (28). The 2018/AHA/ASA recommended that patients with concurrent cardiocerebral infarction within 4.5 h should be given rt-PA at stroke dose, followed by PCI (Class IIa; C) (29). 2021/ESO guidelines suggested that in the uncommon case scenario of an AIS complicating an AMI (< 6 h), alteplase may be administered if there are no other contraindications to intravenous thrombolysis. Mechanical thrombectomy may be an effective therapy in patients with large vessel occlusion and recent myocardial infarction (23). Moreover, successful thrombolytic case of Tenecteplase (TNK) have also been reported (30).

## Conclusion

For patients with AMI-AIS, the clinical manifestations are diverse and complex. There is a lack of studies with large clinical sample data. The treatment includes but is not limited to intravascular thrombectomy, thrombolysis and coronary intervention. Ultimately, individualized plans need to be formulated under multi-disciplinary cooperation. At the same time, clinicians also need to consider the worst clinical outcome before making decisions, and strengthen communication with patients’ families to avoid unnecessary doctor-patient disputes.

## Data availability statement

The raw data supporting the conclusions of this article will be made available by the authors, without undue reservation.

## Ethics statement

Written informed consent was obtained from the individual (s) for the publication of any potentially identifiable images or data included in this article.

## Author contributions

C-HB and Y-BP contributed significantly to analysis and manuscript preparation. CZ and X-MW performed in clinical data collection. C-HB and CZ wrote the manuscript. Y-BP checked and revised the manuscript. All authors contributed to the article and approved the submitted version.

## Abbreviations

AMI: acute myocardial infarction
AIS: acute ischemic stroke
AF: atrial fibrillation
BP: blood pressure
CTA: computed tomography angiography
CAG: coronary angiography
CABG: coronary artery bypass grafting
ECG: electrocardiograph
LAD: left anterior descending artery
LCX: left circumflex artery
NIHSS: national institutes of health stroke scale
NOAC: novel oral anticoagulants
PCI: percutaneous coronary intervention
RCA: right coronary artery
STEMI: ST-segment elevation myocardial infarction
TIMI: thrombolysis in myocardial infarction.
